# Translatability of mouse muscle-aging for humans: the role of sex

**DOI:** 10.1007/s11357-024-01082-7

**Published:** 2024-01-24

**Authors:** Jelle C. B. C. de Jong, Martien P. M. Caspers, Nicole Worms, Nanda Keijzer, Robert Kleemann, Aswin L. Menke, Arie G. Nieuwenhuizen, Jaap Keijer, Lars Verschuren, Anita M. van den Hoek

**Affiliations:** 1 Department of Metabolic Health Research, The Netherlands Organization for Applied Scientific Research (TNO), Leiden, The Netherlands; 2 Human and Animal Physiology, Wageningen University, Wageningen, The Netherlands; 3 Department of Microbiology and Systems Biology, The Netherlands Organization for Applied Scientific Research (TNO), Leiden, The Netherlands

**Keywords:** Sarcopenia, Gender, Frailty, Muscle atrophy, Physical activity

## Abstract

Muscle-aging drives sarcopenia and is a major public health issue. Mice are frequently used as a model for human muscle-aging, however, research investigating their translational value is limited. In addition, mechanisms underlying muscle-aging may have sex-specific features in humans, but it is not yet assessed whether these are recapitulated in mice. Here, we studied the effects of aging on a functional, histological and transcriptional level at multiple timepoints in male and female mice (4, 17, 21 and 25 months), with particular emphasis on sex-differences. The effects of natural aging on the transcriptome of quadriceps muscle were compared to humans on pathway level. Significant loss of muscle mass occurred late, at 25 months, in both male (-17%, quadriceps) and female mice (-10%, quadriceps) compared to young control mice. Concomitantly, we found in female, but not male mice, a slower movement speed in the aged groups compared to the young mice (P < 0.001). Consistently, weighted gene co-expression network analysis revealed a stronger association between the aging-related reduction of movement and aging-related changes in muscle transcriptome of female compared to male mice (P < 0.001). In male, but not female mice, major distinctive aging-related changes occurred in the last age group (25 months), which highlights the necessity for careful selection of age using mice as a muscle-aging model. Furthermore, contrasting to humans, more aging-related changes were found in the muscle transcriptome of male mice compared to female mice (4090 vs. 2285 differentially expressed genes at 25 months, respectively). Subsequently, male mice recapitulated more muscle-aging related pathways characteristic for both male and female humans. In conclusion, our data show that sex has a critical effect on the mouse muscle-aging trajectory, although these do not necessarily reflect sex differences observed in the human muscle-aging trajectory.

**Supplementary Information:**

The online version contains supplementary material available at 10.1007/s11357-024-01082-7.

## Introduction

Sarcopenia is an aging related disease characterized by loss of muscle strength and mass [1, 2] and increases the risk for frailty and mortality in the older population [3, 4]. By the year 2050, the number of people older than 60 is projected to be 2.1 billion, making sarcopenia a critical public health issue [3, 5], that will contribute significantly to healthcare related costs [6]. It is therefore important to develop effective therapeutic interventions that can prevent or reverse the development of sarcopenia. Naturally aged mice are frequently used as a pre-clinical model for testing novel treatments, however, research comparing the mechanisms underlying muscle-aging in mice and humans, and thereby assessing their translatability is limited.

The translatability of the naturally aged mouse should not be assumed, as some factors related to muscle-aging are not conserved across the species. For example, muscle structure is different in mice and humans, since skeletal muscles of mice contain a much higher percentage of type II (fast) myofibers compared to humans [7]. For example, the quadriceps muscle of mice contains almost exclusively type II myofibers [8], while the quadriceps muscle of human contains (on average) a more equally proportioned amount of the two myofiber types [9]. Furthermore, since the lifespan of mice is much shorter, the effects of a sedentary lifestyle or malnutrition have much less time to affect the muscle, which are important predictors for the onset of sarcopenia in humans [10, 11]. Naturally aged mice could therefore exaggerate or fail to mimic mechanisms associated with muscle-aging in humans. Besides, aging-related comorbidities, such as nephropathy, adenocarcinoma, hematopoietic neoplasia or cataracts, are frequently present in aged mice [12], which could go unnoticed and hamper correct interpretation of data.

Aging is accompanied by many changes in different pathways that are important for proper muscle function and content. Subsequently, it is possible that some pathways are conserved across the species, but others are not. For example, cellular senescence, which is a state of irreversible growth arrest due to various aging-related stressors, has been shown to occur in aged skeletal muscle of both humans and mice [13]. Other mechanisms include the development of anabolic resistance, hallmarked by a decreased stimulation of protein synthesis through the mTOR pathways after an anabolic stimulant, such as physical activity [14]. Concomitantly, myofiber size and proportion of type II (fast) myofibers specifically decreases during aging [15], and also here physical activity is an important factor that exerts protective effects [16]. Interestingly, the pathophysiology associated to muscle-aging in humans possibly has sex-specific traits, including, but not limited to the aging-related increase in the proportion of type I (slow) myofibers (more in males) [9], alterations in growth signaling pathways (more in females) [9], increased expression of fibrosis markers (more in females) [17], loss of intramuscular (acyl-)carnitine content (more in females) [18] and frailty associated inflammation (more in females) [19]. Such sex differences should perhaps be taken into account during the pre-clinical stage of development of therapeutic interventions against sarcopenia or frailty. However, it can be questioned whether sex differences observed in human muscle-aging are recapitulated by mice.

Based on these findings, several knowledge gaps were addressed within this study. The primary aim of the study was to assess the translatability of naturally aged mice for human muscle aging and address potential sex differences. We hypothesize that mice recapitulate well-conserved aging related pathways (e.g. cellular senescence) in an age-dependent manner, but lack the recapitulation of pathways influenced by lifestyle (e.g. the effects of sedentary behavior or inadequate nutritional intake on pathways regulating protein synthesis). In addition, we hypothesize that there is a differential impact of aging between the two sexes, and that the translatability of mice is improved when sex-specific comparisons are made. To test these hypotheses, RNA-sequencing was used and gene expression profiles of naturally aged male and female mice were examined and compared to those characteristic of human aging. To do so, we used available muscle samples from well-matched (e.g. age, BMI and frailty status) male and female participants, and compared old vs. young groups for each sex separately [18]. Secondary aims were to assess sex differences in the muscle-aging trajectory of mice and compare those to humans.

## Materials and methods

### Mouse study design

All animal care and experimental procedures were approved by the Ethical Committee on Animal Care and Experimentation (Zeist, The Netherlands; approval reference numbers TNO-440, date: 24th of October 2019 and TNO-478, date: 23th of February 2021), and were in compliance with European Community specification regarding the use of laboratory animals.

C57BL/6 J mice were obtained from Charles River Laboratories (L’Arbresle, France) and all mice were kept on chow maintenance diet (Ssniff Spezialdieten GmbH, Soest, Germany). Mice were housed with at least one littermate in a temperature-controlled room on a 12 h light–dark cycle and had free access to food and water. Four weeks prior to sacrifice, mice were housed individually. After at least two weeks of this acclimatization period, average movement speed was measured every 30 min by means of infrared sensors using TSE PhenoMaster V4.6.2 (TSE Systems, Bad Homburg, Germany) during two consecutive nights (their active time period) and days (their inactive time period). Data of the second day and night were used. One day prior to sacrifice, body weight, lean body mass and grip strength were measured. Grip strength of forepaws was measured using a Grip Strength Meter (TSE Systems, Bad Homburg, Germany). Five trials were performed, the trial with highest and lowest force were excluded and the maximal force of the three remaining trials was averaged. Body weight and lean body mass were measured using an NMR EchoMRI 2-in-1 whole body composition analyzer (Echo Medical Systems LTD, Houston, TX, USA). Mice (n = 12 per group) were sacrificed at timepoints 4 months (16 weeks), 17 months (76 weeks), 21 months (92 weeks) and 25 months (109 weeks) unfasted by gradual fill CO_2_ asphyxiation and hind limb muscles were dissected and weighted. The number of mice was calculated with G*Power software (Heinrich Heine University Dusseldorf, Dusseldorf, Germany) using a one-way ANOVA with a significance threshold of 0.05, a power of 90%, a standard deviation of 15% and a desired minimal difference in our primary parameter (skeletal muscle mass) of 20%. Muscles used for histological analysis were formalin-fixed and paraffin-embedded, while muscles used for transcriptome analysis were frozen in liquid nitrogen.

### Histological analysis

Quadriceps and soleus muscles were isolated, fixed in formalin, cross-sectionally cut at their thickest part and embedded in paraffin. Cross-sections of 5 µm were cut and slides were deparaffinized. For the double staining of Myosin Heavy Chain 7 (MYH7, a slow myofiber specific marker) and laminin (a basal lamina specific marker) epitope was retrieved through a 25 min incubation in 10 mM Sodium Citrate (pH 6) at 95 °C. Endogenous peroxidase activity was masked by a 10 min incubation in 0.3% hydrogen peroxide (v/v) and slides were blocked by a 30 min incubation in 5% normal goat serum in TBS. After blocking, slides were incubated in a mixture of anti-MYH7 (1/200; SAB4200670; Sigma-Aldrich) and anti-Laminin (1/100; NB300-144; Novus Biologicals) in 1% normal goat serum at 4 °C. Next, slides were incubated for one hour at room temperature in a mixture of goat anti-rabbit HRP (1/400; Ab6721; Abcam) and goat anti-mouse alkaline phosphatase (1/400; Ab7069; Abcam) secondary antibodies. Ultimately, slides were developed using a HRP substrate kit (SK-4700; Vector) and alkaline phosphatase substrate kit (Sk-5100; Vector) according to the manufacturer’s instructions. After development, the slides were dehydrated and covered. Slide scans were made at 20 × magnification using a Pannoramic 250 scanner (3D Histech, Budapest, Hungary). Myofiber diameter was quantified by measuring minimal Feret’s diameters using ImageJ, since this parameter is least sensitive to sectioning angle [20]. This analysis was performed in quadriceps using five randomly selected regions of interest (0.18 mm^2^) in 1–2 cross sections per animal. In soleus muscle this was performed for each myofiber type separately using two randomly selected regions of interest. In addition, cross-sections were stained with Sirius Red and muscle fibrosis was identified using ImageJ. Five randomly selected regions of interest (0.18 mm^2^) were used to measure the surface area of collagen. By normalizing for total area, we calculated the percentage of muscle surface area with a positive signal for collagen [21].

### Transcriptomics analysis

RNA extraction was performed as described previously in detail [22]. Total RNA was extracted from quadriceps muscle samples using glass beads and RNA-Bee (Campro Scientific, Veenendaal, The Netherlands). RNA integrity was examined using the RNA 6000 nano Lab-on-a-Chip kit and a bioanalyzer 2100 (Agilent Technologies, Amstelveen, The Netherlands). The NEBNext Ultra II Directional RNA Library Prep Kit (NEB #E7760S/L, New England Biolabs, Ipswich, MA, USA) was used to process the samples. Briefly, mRNA was isolated from total RNA using the oligo-dT magnetic beads. After fragmentation of the mRNA, cDNA synthesis was performed, and cDNA was ligated with the sequencing adapters and amplified by PCR. The quality and yield of the amplicon were measured (Fragment Analyzer, Agilent Technologies, Amstelveen, The Netherlands). The size of the resulting product was consistent with the expected size distribution (a broad peak between 300–500 bp). Clustering and DNA sequencing, using the Illumina NovaSeq6000, was performed according to manufacturer's protocols of service provider GenomeScan B.V. (Leiden, the Netherlands) using a concentration of 1.1 nM of amplicon library DNA and yielding at least 15 million sequencing clusters per sample and 150nt paired-end reads. The genome reference and annotation file Mus_musculus. GRCm38.gencode.vM19 was used for analysis in FastA and GTF format. The reads were aligned to the reference sequence using the STAR 2.5 algorithm with default settings (https://github.com/alexdobin/STAR). Based on the mapped read locations and the gene annotation, HTSeq-count version 0.6.1p1 was used to count how often a read was mapped on the transcript region. These counts served as input for statistical analysis using the DEseq2 package [23]. For pathway analysis, differentially expressed genes were selected (p-value < 0.01) and used as input in Ingenuity Pathway Analysis (IPA, www.ingenuity.com, accessed 2023). Pathways with p-value < 0.01 were considered to be significant. Heatmaps were created using the ‘Pheatmap’ package.

### Weighted gene co-expression network analysis

Weighted gene co-expression network analysis (WGCNA) was used to identify modules of genes that correlate in expression throughout a data set. We used the Bioconductor ‘WGCNA’ package to identify such modules of correlating genes. First, the top 15000 genes with the biggest coefficient of variation were identified for male and female mice separately and used as input. Secondly, using the Soft Thresholding function, a power cutoff was selected that resulted in a scale free topology model fit (signed R^2) of at least 0.8. Third, gene modules were identified by means of hierarchical clustering, using an ‘unsigned’ topological overlap matrix, and modules were merged with a Cut Height of 0.4. Lastly, correlations between modules and night-time movement speed were calculated using the Pearson correlation between the module eigengenes (i.e., first principal component) and movement speed data. Genes that were part of the best correlating module were used as input for pathway analysis using Gene Ontology enrichment analysis (www.geneontology.org), analyzing based on Biological Processes.

### Human reference study characteristics

Aging induced changes in the skeletal muscle transcriptome of mice were compared to those of humans using data obtained in the FITAAL study [18]. Briefly, the FITAAL study is a cross-sectional human study in which 13 male (23.3 ± 0.5 years) and 13 female (22.6 ± 0.5 years) young individuals and 26 elderly males (79.7 ± 0.7 years) and 28 elderly females (80.2 ± 0.6 years) participated. Male and female participants were highly matched in both young and old groups with respect to age and BMI. Males and females were also matched for the Fried frailty score in the old groups. Exclusion criteria included diagnosis with cardiac failure, COPD, anemia, cancer, neuromuscular disorder or dementia, contraindication for muscle biopsy, recent (up to 3 months prior to initiation of the study) significant medical or surgical events or treatment by a medical specialist, current enrolment in another study, intake of carnitine supplements, or usage of several types of medication (e.g., corticosteroids or fibrates). A BMI < 20 kg/m2 or > 25 kg/m2, diagnosis with diabetes mellitus type I and II, and a high frequency of physical exercise (> 4 times a week) served as additional exclusion criteria for young individuals, as did pregnancy or nursing for young female participants. Muscle biopsies were collected as described earlier [18]. 400 m walk test, handgrip strength test and the Short Physical Performance Battery (SPPB) test were performed as validated previously [24–26]. For the 400 m walk time a time limit of 900 s was used, which was given as a final score if participants were unable to finish the 400 m walk test [24]. For the grip strength, the participants were seated in an upward position with their dominant arm positioned in a 90° angle and the average score of three trials was used [25]. For the five time chair stands test, participants were seated on a chair with arms folded across their chest. The time required to finish the fifth stand was used [26]. Frailty score was assed using the frailty score of Fried et al. [27], which is based on weight loss, exhaustion, physical activity, a gait speed test and grip strength. Body composition was measured using a DEXA scan (Hologic Discovery-A, Hologic Inc., Bedford, USA). The study was conducted according to the declaration of Helsinki, was approved by the medical ethical committee of Wageningen University (METC nr. 16/20), and was registered in the Dutch Trial Register (NTR6124). All participants provided written informed consent prior to enrolment. An overview of subject characteristics can be found in Table 1.

**Table 1:** Participant characteristics. Presence of different letters (a-d) across a row indicate significant differences among respective groups. Values are averages ± SEM

	Males		Females	
	Young (n = 13)	Old (n = 28)	Young (n = 13)	Old (n = 26)
Age (years)	23.3 ± 0.5^a^	79.7 ± 0.7^b^	22.6 ± 0.5^a^	80.2 ± 0.6^b^
Weight (kg)	76.2 ± 2.5^a^	81.0 ± 2.1^a^	63.9 ± 1.7^b^	68.9 ± 10.3^b^
BMI (kg/m^2^)	22.5 ± 0.3^a^	26.4 ± 0.8^b^	22.2 ± 0.5^a^	26.2 ± 0.6^b^
Body fat (%)	16.9 ± 0.9^a^	25.3 ± 0.9^b^	29.1 ± 1.1^c^	35.7 ± 0.8^d^
Lean mass (%)	79.3 ± 0.9^a^	71.3 ± 0.8^b^	67.3 ± 1.0^c^	61.4 ± 0.8^d^
BMC (%)	3.7 ± 0.1^a^	3.3 ± 0.1^b^	3.6 ± 0.1^a^	2.9 ± 0.1^b^
Handgrip strength (kg)	-	32.0 ± 1.4^a^	-	22.2 ± 1.1^b^
400 m walk test (s)	-	323.3 ± 7.6^a^	-	393.9 ± 31.0^b^
SPPB (points)	-	10.0 ± 0.4^a^	-	8.9 ± 0.6^a^
Fried frailty score	-	0.5 ± 0.1^a^	-	0.7 ± 0.2^a^

### Statistical analysis

Statistical differences between groups (of data other than transcriptomics data) were determined using IBM SPSS Statistics 25 (IBM, NY, USA). Normality of data was tested using a Kolmogorov–Smirnov and Shapiro–Wilk test. To test for the effects of sex and age, and whether there is an interaction between these two factors, a two-way ANOVA was performed with sex and age as independent factors. If data was not normally distributed, then data was log transformed prior to the two-way ANOVA to approach normal distribution. In addition, to gain insight in the within-sex effects of aging, a Tukey’s test for post-hoc analysis was performed for each sex separately. Between-sex comparisons were not included in the post-hoc analyses, since we are not trying to address differences in males and females at a certain timepoint, but only the effects of aging and the interaction with sex. If data was not normally distributed, then a Kruskal–Wallis test followed by a Mann–Whitney U test was used for post-hoc analysis (an overview is provided in supplementary Table 1). Differences in data distribution across categorial groups were tested using a Chi-squared test. A P-value < 0.05 was considered statistically significant, two-tailed P-values were used and all values are displayed as mean ± SEM.

## Results

### Aging trajectories of movement speed and body fat mass are highly sex-specific in mice

**Fig. 1:**
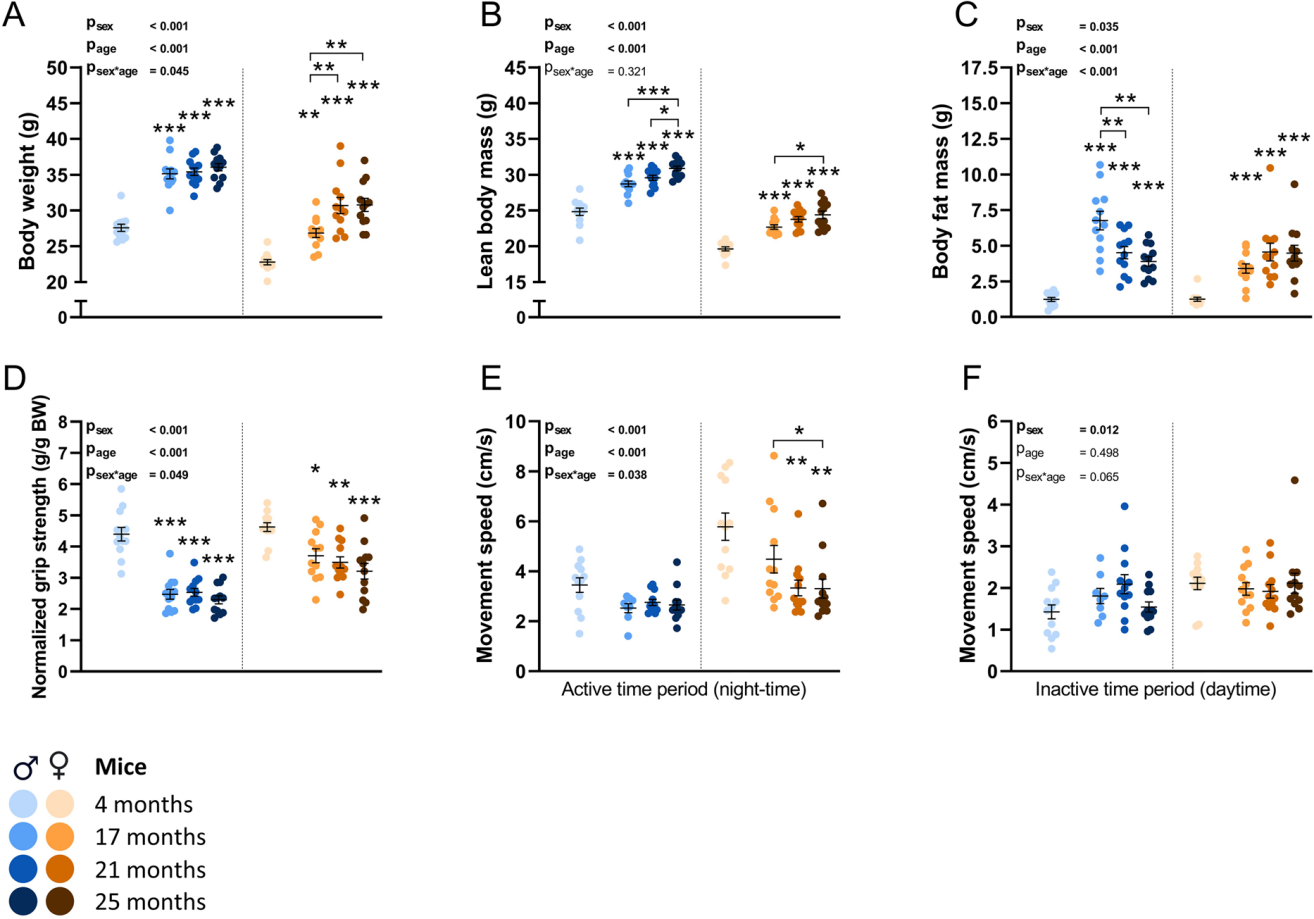
The effects of aging on body composition, grip strength and movement speed in male and female mice. (A) Body weight, (B) lean body mass and (C) body fat mass. (D) Grip strength of forepaws normalized for body weight. (E) Movement speed during night-time and (F) day-time. Values represent mean ± SEM, *p < 0.05, **p < 0.01, ***p < 0.001

To map the aging trajectories of male and female mice, mice of both sexes were terminated and dissected at different timepoints (4, 17, 21 and 25 months of age), prior to which a variety of functional measurements were performed. Body weight, in both sexes, was significantly higher in all aged groups compared to the 4 months old group (Fig. 1A, P_age_ < 0.001). In females, body weight was significantly higher in 21 and 25 months old mice compared to 17 months old mice as well (P < 0.01). In line with body weight, lean body mass was significantly higher in all aged groups compared to the 4 months old groups of both sexes (Fig. 1B, P_age_ < 0.001). Lean body mass was significantly higher in 25 months old male mice compared to 17 (P < 0.001) and 21 (P < 0.05) months old mice, indicating a lack of aging associated loss of lean body mass. In female mice, lean body mass was significantly higher in 25 months old mice compared to 17 months old mice as well (P < 0.05). Interestingly, body fat mass data displayed highly sex-specific aging trajectories (Fig. 1C), as indicated by the interaction between sex and age as well (P_sex*age_ < 0.001). Aged male mice initially gained body fat mass at 17 months compared to 4 months (P < 0.001), while at later ages mice had significantly lower amounts of body fat mass compared to the 17 months old group (P < 0.01). Contrastingly, females also initially significantly gained body fat mass at 17 months (P < 0.001), but older groups did not lose body fat mass and even showed a trend towards further gain of fat mass at 25 months old compared to 17 months old mice (P = 0.06). Lean body and fat mass data expressed as relative data are available in supplementary Fig. 1. In addition, food intake was not lower in all aged groups compared to the 4 months old mice (supplementary Fig. 1). Grip strength was lower in all aged groups of both sexes compared to the 4 months old groups (Fig. 1D, P_age_ < 0.001). Movement speed was measured during day and night-time separately (Fig. 1E-F). Strikingly, in male mice no significant differences in night-time movement speed were found amongst the different age groups (Fig. 1E). Contrastingly, in female mice, at 21 and 25 months night-time movement speed was significantly lower compared to 4 (P < 0.01) and 17 months old mice (P < 0.05), indicating a more pronounced decrease of movement speed in female mice compared to male mice during aging, as also suggested by the significant interaction between sex and age (P_sex*age_ < 0.05). No differences in day-time movement speed were found during aging (Fig. 1F, P_age_ = 0.498). Similar effects of sex and age were found in regards to daily ambulatory distance (supplementary Fig. 1).

### Quadriceps was the only muscle group to display significant aging-related loss of muscle mass in both sexes

**Fig. 2:**
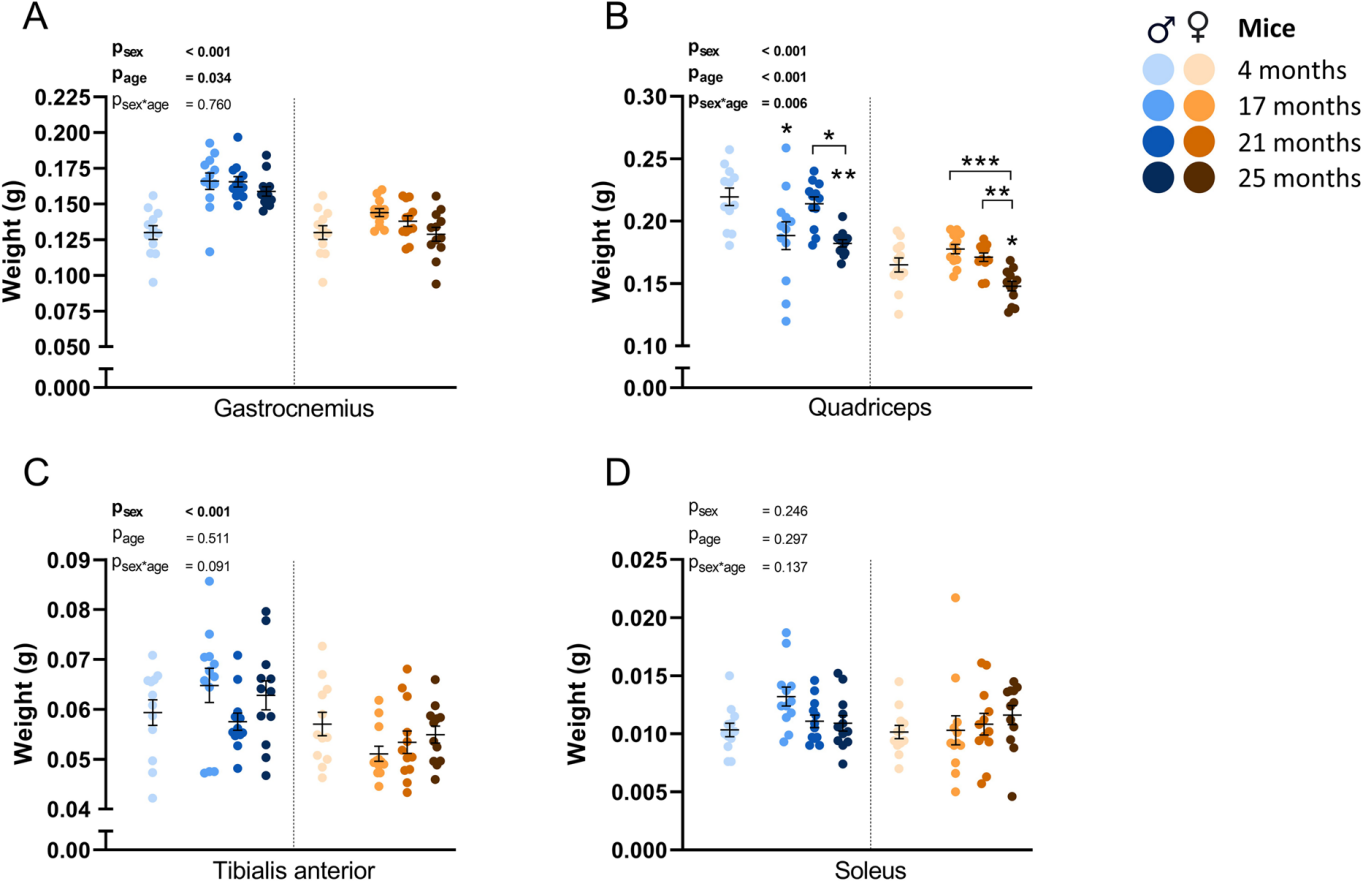
Weights of muscle groups directly after dissection in male and female mice. (A) Gastrocnemius, (B) quadriceps, (C) tibialis anterior and (D) soleus muscle group weight are given. Values represent mean ± SEM, *p < 0.05, **p < 0.01, ***p < 0.001

Muscle groups were weighed directly after the dissections. A significant effect of age was found on the weight of the gastrocnemius muscle (P_age_ < 0.05), but no statistically significant differences were found in the post-hoc analysis. Age also significantly affected quadriceps muscle weight (P_age_ < 0.001), and post-hoc analysis revealed a significant lower weight at 17 months (P < 0.05) and 25 months (P < 0.01) old male mice compared to 4 months old male mice. In female mice, a significant loss of muscle mass was detected at 25 months of age (P < 0.05) compared to the 4 months old group (Fig. 2B). No significant effects of age were measured in tibialis anterior (Fig. 2C, P_age_ = 0.511) and soleus (Fig. 2D, P_age_ = 0.297) muscle groups.

### Aging related increase in percentage of type I myofibers occurred later in male compared to female mice

**Fig. 3:**
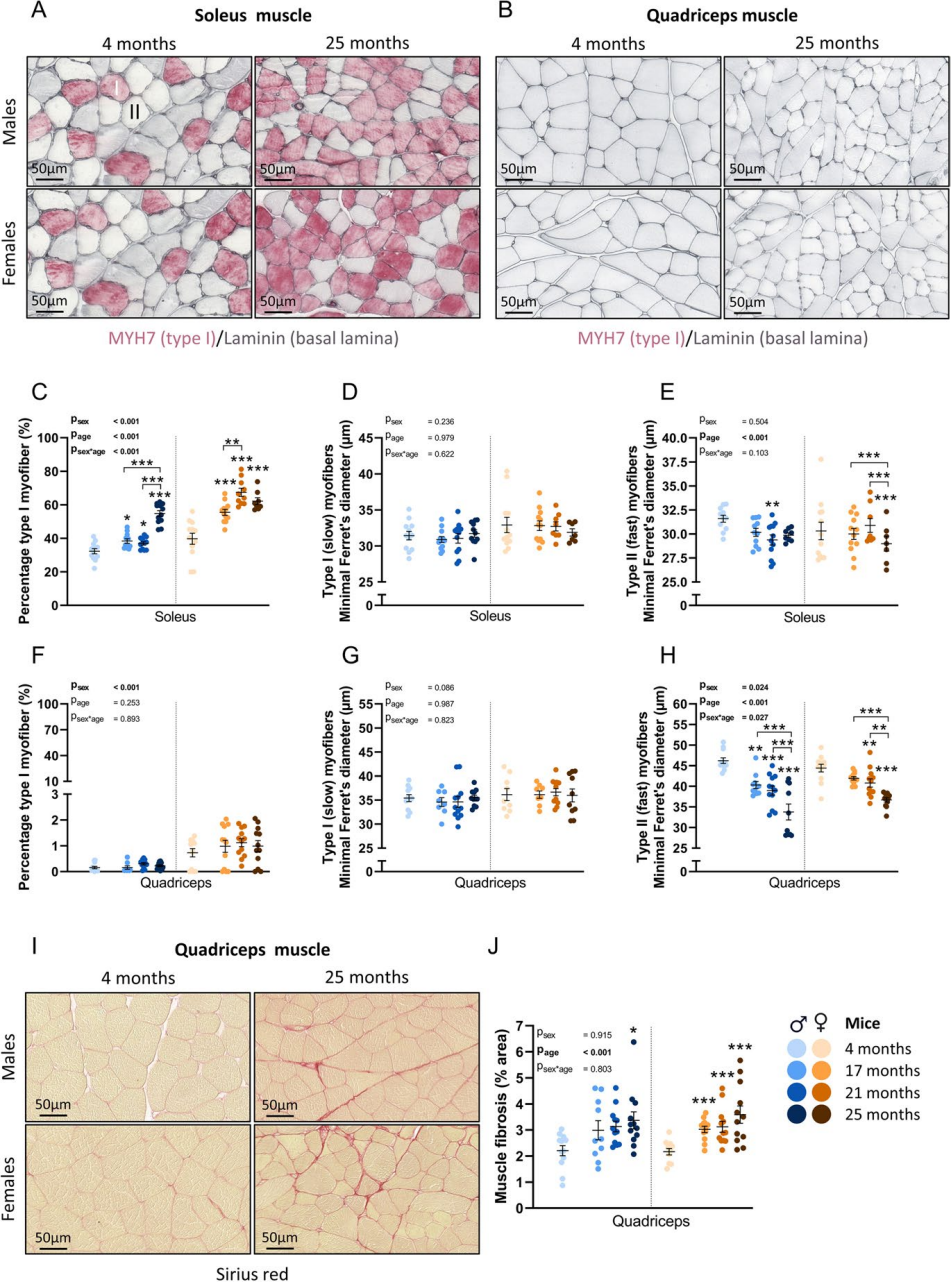
Histological characterization of soleus and quadriceps muscles in male and female mice. (A-B) Representative pictures of immunohistochemical stainings used to identify type I (slow) and II (fast) myofibers. (C) Percentage of type I myofibers in soleus muscle. (D) Minimal Ferret’s diameter of type I myofibers and (E) type II myofibers in soleus muscle. (F) Percentage of type I myofibers in quadriceps muscle. (G) Minimal Ferret’s diameter of type I myofibers in quadriceps muscle. (H) Minimal Ferret’s diameter of type II myofibers in quadriceps muscle. (I) Representative pictures of muscle fibrosis (Sirius red) in quadriceps muscle. (J) Muscle fibrosis, quantified by % of total area in quadriceps muscle. Values represent mean ± SEM, *p < 0.05, **p < 0.01, ***p < 0.001

Soleus and quadriceps muscles were used for immunohistological stainings to identify slow (type I) and fast (type II) myofibers (Fig. 3A-B). The percentage of type I myofibers was significantly higher in the soleus muscle of all aged groups compared to the 4 months old group in both sexes (Fig. 3C, P_age_ < 0.001). However, the percentage of type I myofibers was primarily higher at the age of 25 months in male mice (P < 0.001 compared to 17 and 21 months). In females, the percentage of type I myofibers was higher in 17 months compared to 4 months old mice (P < 0.001), and even higher in 21 months old mice compared to 17 months old mice (P < 0.01). No further increase was observed at 25 months of age in females, differing from males as also indicated by the significant interaction between sex and age (P_sex*age_ < 0.001). In soleus muscle, the diameter of type I myofibers of both sexes did not change during aging (Fig. 3D, P_age_ = 0.979), whereas the diameter of type II myofibers did change with aging (Fig. 3E, P_age_ < 0.001) and was smaller in some of the older groups compared to the 4 months old groups in both sexes (P < 0.01 in 21 months old males and P < 0.001 in 25 months old females compared to 4 months old mice). In quadriceps muscles, a very small amount of type I myofibers were detected (ranging from 0 to 2%), of which the percentage did not change during aging (Fig. 3F, P_age_ = 0.253). The diameter of these type I myofibers also did not change during aging (Fig. 3G, P_age_ = 0.987). In type II myofibers of the quadriceps muscle age had a significant effect (Fig. 3H, P_age_ < 0.001), and post-hoc analysis revealed a significantly smaller diameter at the ages of 17 and 21 months (P < 0.01 and P < 0.001, respectively) compared to 4 months old male mice. At 25 months the average diameter was even smaller compared to 21 months old male mice (P < 0.001). In female mice, type II myofibers were smaller at 21 and 25 months of age compared to 4 months old mice (P < 0.01 and P < 0.001, respectively), and also here the diameter of 25 months old mice was significantly smaller compared to 21 months old mice (P < 0.01). Noticeably, in type II myofibers, significant differences were found at earlier ages in the quadriceps muscle compared to the soleus muscle, indicating an earlier or more pronounced loss of myofiber size in quadriceps compared to soleus muscle. Frequency graphs of diameter data of both myofiber types in the quadriceps and soleus muscles are attached as supplementary Fig. 2. Muscle fibrosis was measured in quadriceps muscle (Fig. 3I) and was significantly affected by age (P_age_ < 0.001). Muscle fibrosis was higher in 25 months old male mice compared to 4 months old male mice (Fig. 3J, P < 0.05). A tendency for more muscle fibrosis was found at 21 months as well (P = 0.09). In female mice, significant muscle fibrosis was detected in all aged groups compared to the 4 months old group (all P < 0.001).

### Aging effects the muscle transcriptome of male, but not female mice, distinctively at 25 months of age

**Fig. 4:**
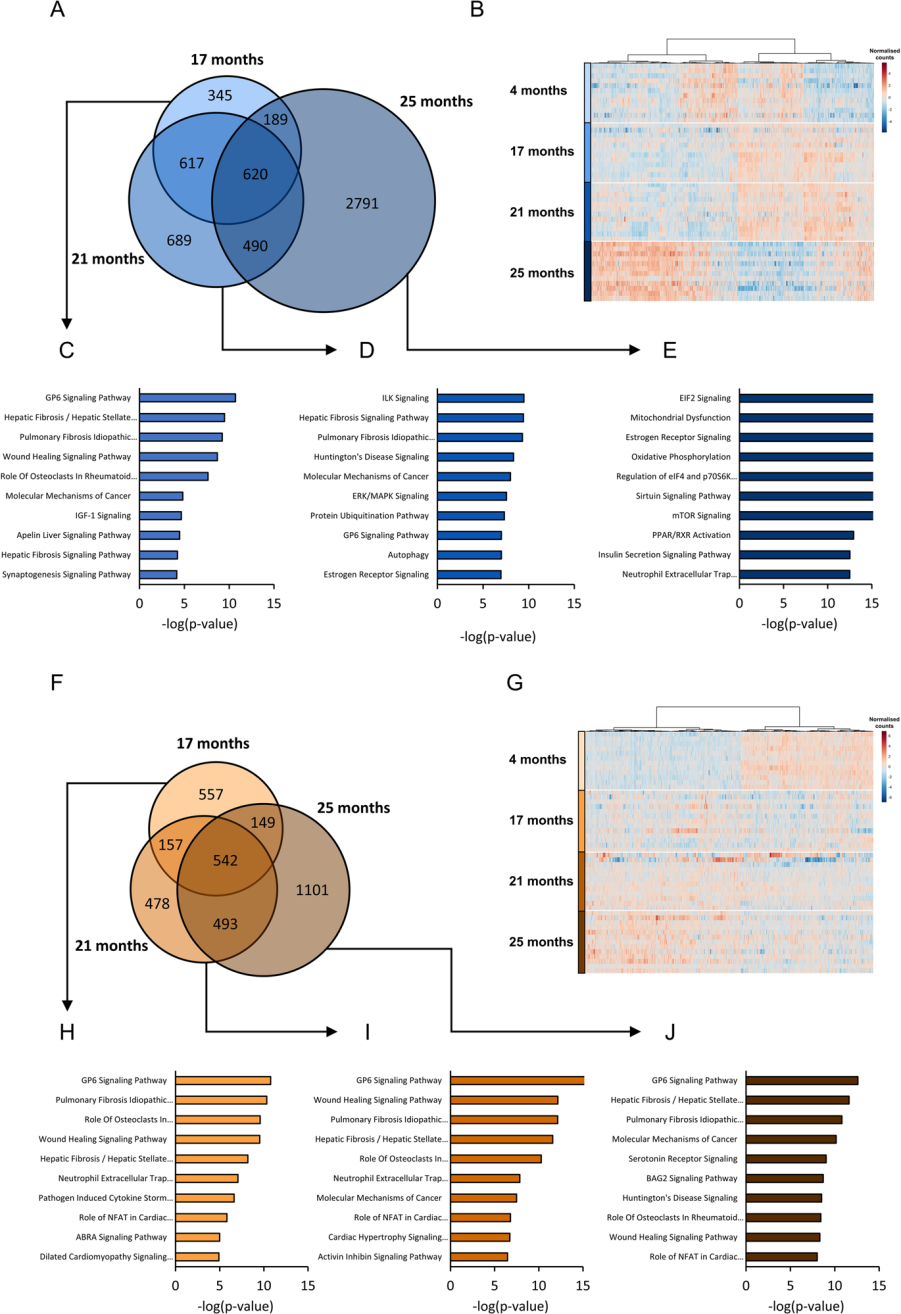
Overview of quadriceps muscle RNA-seq data. (A) Venn diagrams displaying unique and shared DEGs between the aged male groups (17, 21 and 25 months old groups, each compared to the 4 months old group). (B) Heatmap displaying normalized expression of genes differentially expressed in at least one of the aged male groups. A red color indicates a relatively high number of counts, and a blue color indicates a relatively low number of counts for the respective gene. (C) Top 10 pathways of male mice at 17 months, (D) 21 months and (E) 25 months, based on all genes that were differentially expressed compared to the 4 months old mice. (F) Venn diagrams displaying unique and shared DEGs between the aged female groups (17, 21 and 25 months old groups, each compared to the 4 months old group). (G) Heatmap displaying normalized expression of genes differentially expressed in at least one of the aged female groups. (C) Top 10 pathways of female mice at 17 months, (D) 21 months and (E) 25 months, based on all genes that were differentially expressed compared to the 4 months old mice

Since the largest aging effects were found on the weight of the quadriceps muscle group, this muscle group was used for RNA-sequencing to gain insight in the pathways associated to muscle-aging. Aged groups (17, 21 and 25 months) were compared to the young (4 months) group for each sex separately and differentially expressed genes (DEGs) were compared (Fig. 4A).

In male mice, each aged group displayed a number of unique DEGs, and 620 DEGs were shared between all aged groups. Strikingly, at 25 months old, a large set of unique genes were differentially regulated (n = 2791), indicating large aging effects at the age of 25 months, which did not yet occur at 17 or 21 months of age (Fig. 4B). Differences in gene expression between the mice of the different groups were visualized using principle component analysis as well (Suppl. Figure 3A). All DEGs of each aged group were used as input for pathway analysis to reveal which biological processes were affected. Top 10 pathways at 17 months of age included pathways related to collagen (e.g. GP6 signaling pathway and hepatic fibrosis hepatic stellate cell activation), growth signaling (IGF-1 signaling, molecular mechanisms of cancer) and the neuromuscular junction (synaptogenesis signaling pathway) (Fig. 4C). At 21 months, top 10 pathways included pathways related to extracellular signaling (ILK signaling and ERK/MAPK signaling), collagen (e.g. GP6 signaling pathway), protein breakdown (protein ubiquitination and autophagy) and estrogen signaling (estrogen receptor signaling) (Fig. 4D). At 25 months, top 10 pathways included key aging related pathways related to (mitochondrial) metabolism (e.g. sirtuin signaling and oxidative phosphorylation), protein synthesis (e.g. mTOR signaling and regulation of eIF4 and p70S6k) and growth signaling (e.g. insulin secretion signaling pathway) (Fig. 4E). The results of this pathway analysis reveal that the used age of the naturally aged mouse model has critical consequences on which molecular alterations are present in the skeletal muscle tissue of the model. Major changes occurred in male mice primarily in the last age group (25 months).

In female mice, all aged groups (17, 21 and 25 months) were compared to the young (4 months) old group. In all aged groups compared to the young group, 542 genes exhibited differential expression, and at 25 months of age, 1101 unique DEGs were identified (Fig. 4F). Genes were clearly separated into two clusters, one consisting out of genes that were upregulated and one cluster containing genes that were downregulated during the muscle-aging trajectory (Fig. 4G). Differing from male mice, the distinct gene expression profile at 25 months was not present in the female mice. The different patterns were also reflected by a principal component analysis (Suppl. Figure 3B). Pathway analysis revealed that the top 10 pathways at 17 months of age included many pathways related to collagen (e.g. GP6 signaling pathway and hepatic fibrosis hepatic stellate cell activation) and immune cell signaling (e.g. pathogen induced cytokine storm signaling pathway and neutrophil extracellular trap signaling pathway) (Fig. 4H). This signature was part of the top 10 pathways in female mice in the older groups as well, as in the 21 and 25 months old mice again primarily pathways related to collagen and immune cell signaling were found (Fig. 4I-J). In fact, the top regulated pathway (GP6 signaling pathway) was the top regulated pathway in all aged groups. At 25 months of age pathways related to cellular growth (e.g. molecular mechanisms of cancer) and nerve cell signaling (e.g. serotonin receptor signaling) were present in the top 10 regulated pathways as well (Fig. 4J).

### The female muscle-aging trajectory is affected more by the aging-related reduction of movement compared to male mice

**Fig. 5:**
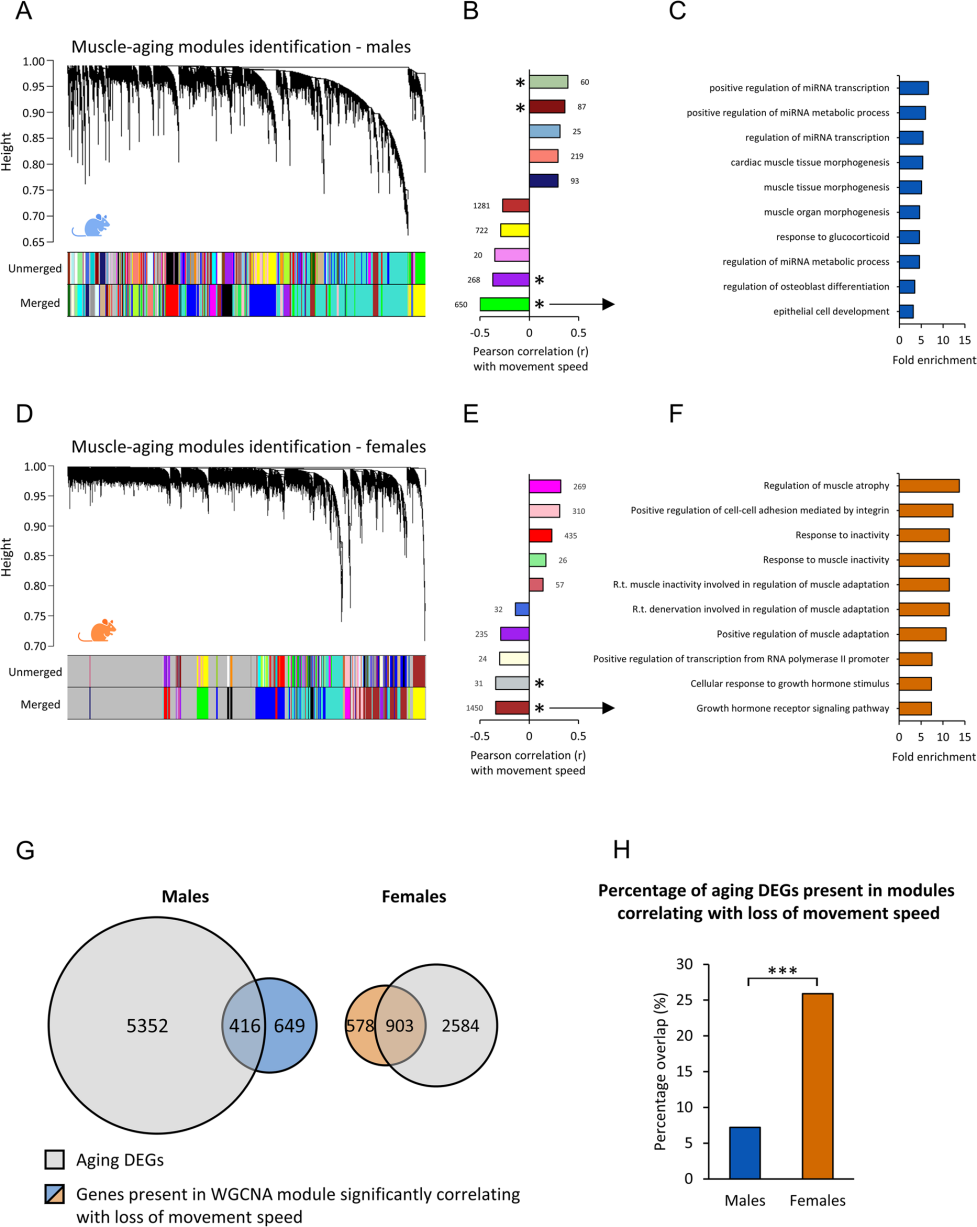
Correlation analysis between gene modules (identified by WGCNA) and aging related loss of movement speed for each sex separately. (A) WGCNA was used to identify modules in the male mouse groups. (B) Correlation analysis between the identified modules and night-time movement speed in male mice. Numbers behind bars indicate number of genes present in the module. (C) Pathway analysis of the best correlating module, the green module as indicated by the arrow. (D) WGCNA of gene expression data of female mice. (E) Correlation analysis between the identified modules and night-time movement speed in female mice. Numbers behind bars indicate number of genes present in the module. (F) Pathway analysis of the best correlating module, the brown module as indicated by the arrow. (G) Venn diagrams indicating the overlap between (1) genes that were significantly differentially expressed in at least one of the aged groups (compared to the 4 months old group) and (2) genes that were part of a module significantly correlating with aging related loss of movement speed. (H) The percentage of aging DEGs that were also part of a module significantly correlating with aging related loss of movement speed. * symbols indicate a p < 0.05 for the correlation between a module’s eigengene (i.e., first principal component) and night-time movement speed

Physical activity has been shown to be strongly associated with muscle-aging, and can even reverse aging-related changes in skeletal muscle [28, 29]. Since the aged female mice, but not the aged male mice, displayed a slower night-time movement speed (Fig. 1E), we were interested in assessing the number of aging related DEGs that correlate with, and possibly are influenced by, aging related reduction of movement speed. Weighted gene co-expression network analysis (WGCNA) was performed to cluster genes into modules, and highly related modules were merged. Subsequently, correlation analysis was performed to identify modules significantly correlating with night-time movement speed (Fig. 5A-B). Pathway analysis was performed on the module with the best correlation, which was the green module in male mice containing 650 genes. Pathways related to, among others, mRNA processing and muscle morphogenesis were identified (Fig. 5C).

In female mice WGCNA analysis was performed as well, followed by a correlation analysis between the first principal component of the modules (i.e., module eigengenes) and night-time movement speed (Fig. 5D-E). Noticeably, the best correlating module in female mice contained 1450 genes, which were more genes than the best correlating module found in male mice. Interestingly, using the genes of this module for pathway analysis, pathways related to muscle atrophy, response to muscle inactivity, denervation and growth hormone signaling were found (Fig. 5F).

To quantify and assess the role of movement speed within the aging related changes observed in the muscle transcriptome, the overlap between aging DEGs and genes in significantly correlating modules was measured (Fig. 5G). Strikingly, in female mice many more aging DEGs were also present in the modules significantly correlating with the observed aging-related slower movement speed compared to the male mice (26% in females vs. 7% in males). This distribution was significantly different in male and female mice (P < 0.001), as tested using a Chi-squared test, indicating a larger role of aging related decrease of movement speed in muscle-aging in female mice compared to male mice (Fig. 5H).

### A sex-specific overview of shared and non-shared pathways related to muscle-aging in mice and humans

**Fig. 6:**
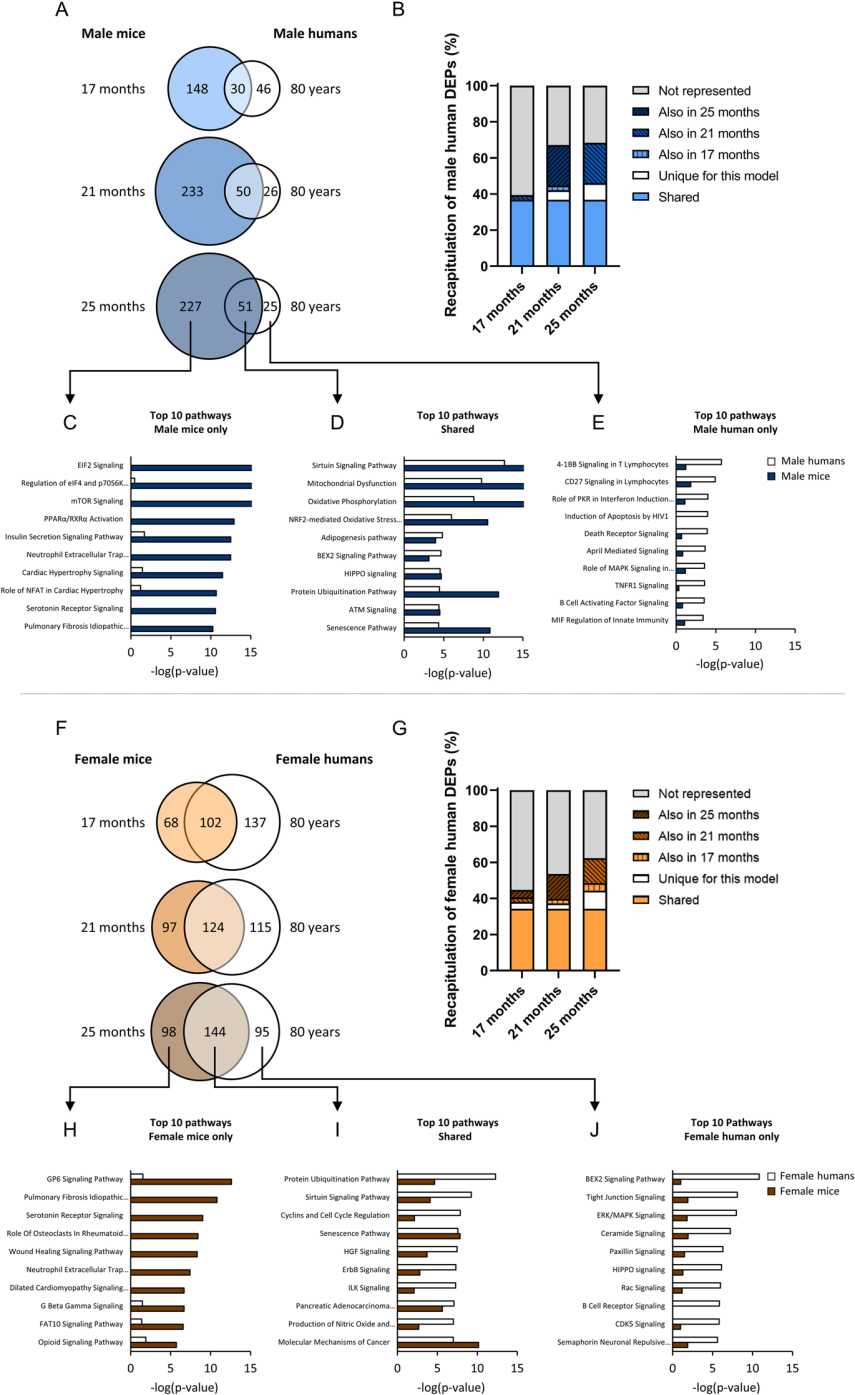
Overview of sex-specific comparisons made between muscle-aging pathways in mice vs. humans. For human reference, data of the FITAAL study was used [9]. (A) Venn diagrams displaying the overlap of pathways that were regulated in male mice (at different ages) and in male humans. (B) Bar graph indicating the percentage of male human pathways also differentially expressed in male mice at different ages. (C) Top 10 pathways differentially regulated in 25 months old male mice but not in male humans. (D) Top 10 pathways differentially regulated in both 25 months old male mice and male humans. (E) Top 10 pathways differentially regulated in male humans but not in 25 months old male mice. (F) Venn diagrams displaying the overlap of pathways that were regulated in female mice (at different ages) and in female humans. (G) Bar graph indicating percentage of female human pathways also differentially expressed in female mice at different ages. (H) Top 10 pathways differentially regulated in 25 months old female mice but not in female humans. (I) Top 10 pathways differentially regulated in both 25 months old female mice and female humans. (J) Top 10 pathways differentially regulated in female humans but not in 25 months old female mice

To assess the translatability of naturally aged mice for human muscle-aging, differentially expressed pathways in mice were compared to the pathways differentially expressed in humans, for each sex separately. In both species the same muscle, the quadriceps/vastus lateralis, was used for RNA-sequencing. The data used as human reference here originates from the FITAAL study [9, 18]. Uniquely, both male and female participants were recruited during the FITAAL study, and the male and female participants were matched (e.g. in regards to age, BMI and frailty status), thereby isolating the effect of sex on the muscle-aging trajectory. In male humans, 76 pathways were found to be differentially expressed. Venn-diagrams were used to visualize the overlap between the differentially expressed pathways identified in male mice and male humans (Fig. 6A). The number of human pathways that were also differentially expressed in mice was higher in 21 months old mice (n = 50) compared to 17 months old mice (n = 30), but only one additional pathway was recapitulated by mice aged 25 months compared to mice aged 21 months (n = 51). In percentages, 39%, 67% and 68% of the male human muscle-aging pathways were recapitulated by male mice aged 17, 21 or 25 months, respectively, with substantial overlap between the different ages (Fig. 6B). Noticeably, many of the human pathways recapitulated by the 21 and 25 months old mice were shared. To gain more insight in the overlap of pathways regulated in male mice and male humans, top shared pathways, or pathways uniquely regulated in one of the species were examined. Top pathways regulated in male mice but not in male humans consisted primarily of pathways related to muscle protein synthesis, which were all upregulated in male mice (e.g. EIF2 signaling, regulation of eIF4 and p70S6K signaling, mTOR signaling and insulin secretion signaling) (Fig. 6C). Pathways regulated in both male humans and mice consisted of classical aging pathways known to be conserved across species [30], including sirtuin signaling, oxidative phosphorylation, oxidative stress and senescence (mixed up- or downregulation, Fig. 6D). Pathways that were affected in humans but not recapitulated by aged male mice primarily included pathways related to immune cell signaling, which were either downregulated or had no direction of regulation in male humans (e.g. 4-1BB signaling in T lymphocytes and CD27 signaling in lymphocytes) (Fig. 6E).

Similar comparisons were performed for the female datasets. In female humans, 239 pathways were found to be differentially expressed. Venn diagrams (Fig. 6F) revealed that the number of pathways overlapping with human muscle-aging was the lowest in the 17 months old group and the highest in the 25 months old group. In percentages, 45%, 54% and 62% of the female human muscle-aging pathways were recapitulated by female mice aged 17, 21 or 25 months, respectively (Fig. 6G). Top pathways regulated in 25 months old mice but not in humans contained pathways among others related to collagen, which were downregulated in female mice (e.g. GP6 signaling pathway and pulmonary fibrosis idiopathic signaling pathway) (Fig. 6H). Top regulated pathways shared between female mice and humans contained classical aging pathways, including protein ubiquitination pathway, sirtuin signaling pathways, pathways related to cellular senescence and differentiation (e.g. cyclins and cell cycle regulation and senescence pathway) and oxidative stress (mixed up- or downregulation, Fig. 6I). Top pathways regulated in female humans but not in female mice were related to among others the extra cellular matrix, which were either downregulated or had no direction of regulation in female humans (e.g. paxillin signaling and tight junction signaling).

**Fig. 7:**
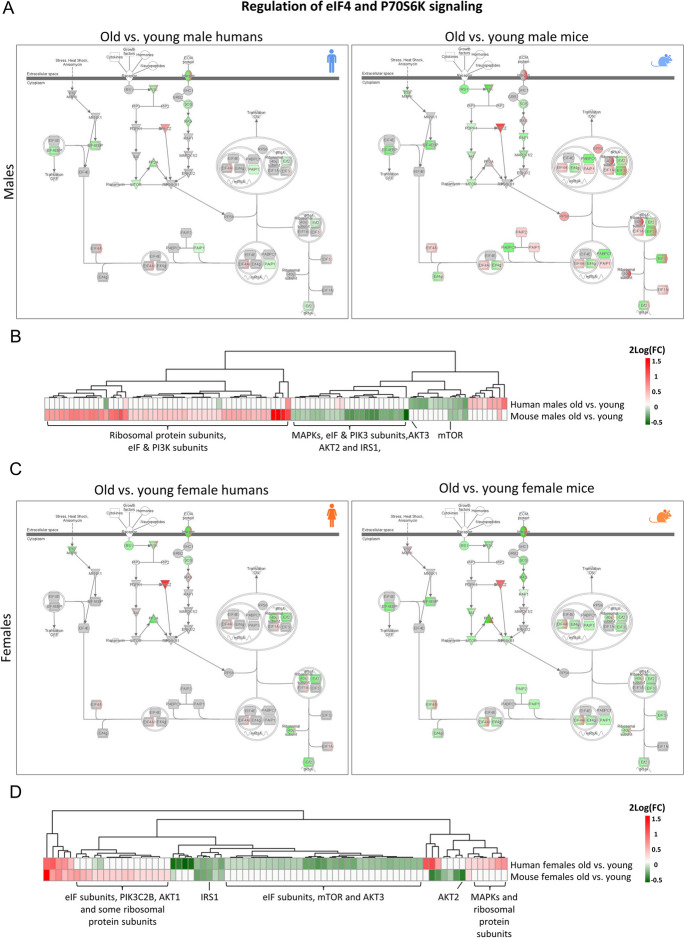
A comparison of aging related changes in the eIF4 and P70S6K signaling pathway between mice and humans for each sex separately. (A) A schematic overview of the changes in the expression of the involved genes in old vs. young male humans and mice. (B) A heatmap with hierarchical clustering of genes significantly differentially expressed in old vs. young male humans and/or mice. The same schematic overview (C) and heatmap (D) were created using the results of the comparison between old and young female humans and mice as input. In all figures a red color indicates an increased expression, and a green color indicates a decreased expression in the aged group compared to the young group. For mice, data belonging to the comparison between the oldest group (25 months) with the youngest group (4 months) was used

Regulation of eIF4 and P70S6k is one of the pathways found to not be consistently affected by aging in mice and humans, and plays an important role in muscle protein synthesis [31]. Genes regulated in this pathway were visualized (Fig. 7A) and a heatmap using 2log(FC) data of significantly regulated genes revealed highly species-specific clusters of genes being regulated (Fig. 7B), including ribosomal protein subunits (upregulated in old vs. young male mice only), eIF & PI3K subunits (some up and some down regulated in old vs. young male mice only) and important signaling proteins such as IRS1 and AKT2 (downregulated in old vs. young male mice only). Aging related changes in the same pathway were visualized using female data (Fig. 7C), and also here species-specific clusters of regulated genes were found. Contrasting to male mice, PI3K and eIF subunits, and important (signaling) proteins including mTOR and AKT3 were not downregulated in female mice, but were down regulated in female humans. This latter finding suggests male mice better recapitulate changes in the eIF4 and P70S6K signaling pathway found in female humans, compared to female mice.

### Human sex differences in aging-related muscle transcriptome are not necessarily reflected by mice

**Fig. 8:**
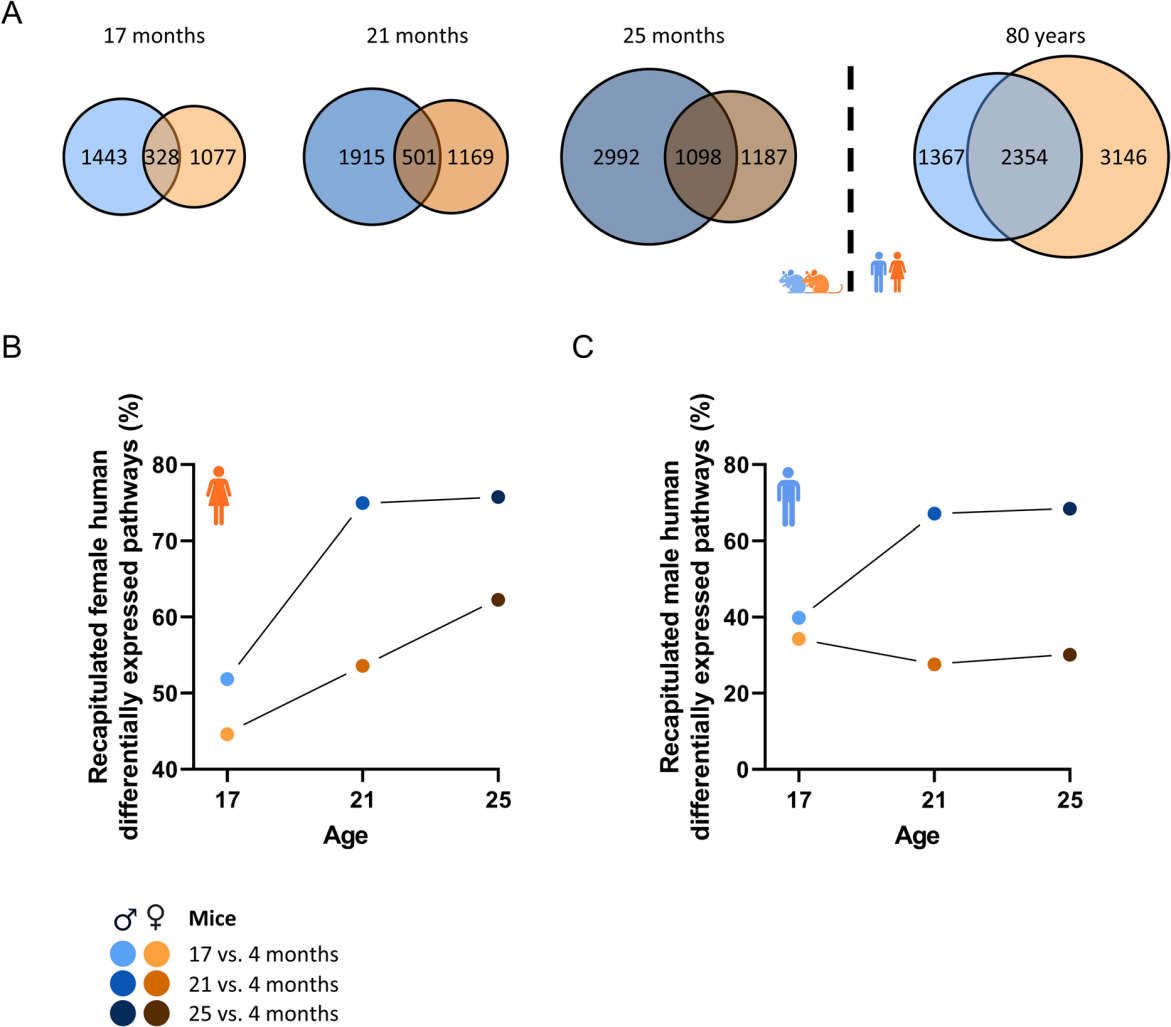
A comparison of sex differences in humans and mice, focusing on aging related perturbations in the transcriptome of quadriceps muscle. (A) Venn diagrams displaying the number of genes significantly regulated in both or only one of the sexes in mice at 17, 21 and 25 months compared to 4 months old mice. On the right side of the dashed line, the number of genes significantly regulated in both or only one of the sexes in old vs. young humans is displayed. (B) The percentage of pathways differentially expressed in old vs. young human females, that were also differentially expressed in old vs. young mice at different ages for both male and female mice. (C) The percentage of pathways differentially expressed in old vs. young human males, that were also differentially expressed in old vs. young mice at different ages for both male and female mice

In this study we found more changes in the skeletal muscle transcriptome of male mice compared to female mice. Strikingly, this is in contrast to what we recently found in multiple human studies. Using four publicly accessible GEO data sets, we consistently found that more genes are differentially expressed in old vs. young female humans compared to old vs. young male humans [9], consistent with the FITAAL study (Fig. 8A). Next, we questioned whether the general assumption, i.e. that female mice better reflect female humans and male mice better reflect male humans, is correct. To assess this, the percentage of pathways that were differentially regulated in female humans and also in male or female mice was calculated for the different age groups (Fig. 8B). Noticeably, male mice recapitulated more pathways that were regulated in old vs. young female humans compared to female mice. This finding was consistent at all age groups of mice, indicating that female mice are not necessarily a better model for female human muscle-aging compared to male mice. Lastly, as expected, male mice also recapitulated more pathways that were regulated in old vs. young human males compared to female mice (Fig. 8C).

## Discussion

The aim of this study was to assess the translatability of naturally aged mice and to investigate potential sex-differences in skeletal muscles during the process of aging in mice. Several sex differences were identified in the muscle-aging trajectories of mice. Movement speed was significantly slower in female, but not male aged mice compared to young control mice. Consistently, WGCNA revealed a stronger association between the decrease of movement and aging-related changes in the skeletal muscle transcriptome of female mice compared to male mice. Together, this indicates a more important role of the decrease of movement speed in skeletal muscle-aging in female mice compared to male mice. Furthermore, we observed in male but not in female mice, major aging-related changes primarily in the last age group (25 months), suggesting the existence of a ‘tipping point’ in muscle-aging of male mice specifically, and highlighting the necessity for careful selection of age using naturally aged male mice as a muscle-aging model. In addition, male mice displayed a larger amount of genes being regulated during aging compared to female mice. This is in contrast to what is observed in humans, as we recently observed using multiple GEO datasets the opposite effect in humans, namely a larger amount of differentially expressed genes in old vs. young females, compared to old vs. young males. In addition, sex-specific comparisons were made with human data, and revealed that a significant amount, but not all pathways related to muscle-aging were recapitulated by mice. For example, pathways related to protein synthesis were disproportionally regulated in male mice compared to male humans (perhaps due to a higher proportion of type II myofibers in mouse quadriceps muscle), while well-known aging-related pathways involving e.g. sirtuin signaling, oxidative phosphorylation, oxidative stress and cellular senescence were conserved across the species. All in all, these findings provide novel insights in sex differences in the muscle-aging trajectories of mice, and suggest that the sex-differences observed in mice do not necessarily reflect sex-differences observed in human muscle-aging.

Aged female, but not male mice displayed a significantly slower movement speed compared to young control mice, which is a finding that is in line with previous studies [32, 33]. However, whether this is associated to potential female-specific changes in the skeletal muscle trajectory was not yet known. Using WGCNA, we now confirmed that indeed a larger part of the muscle-aging signature is associated with aging related decrease of movement speed in female mice compared to male mice. Noticeably, the highest ranking pathways (based on their p-values) found in the best correlating module of female mice were mostly related to (muscle) inactivity and atrophy, confirming the relationship between muscle physical activity and mechanisms underlying muscle atrophy. These findings are relevant, as these have multiple implications for the use of female naturally aged mice in research. The most important implication perhaps is that when naturally aged mice are used to study sex-differences in muscle-aging, potential sex-differences do not necessarily originate from effects of sex per se, but possibly rather from a reduction of physical activity in female mice specifically. On the other hand, if researchers would like to study the effects of the aging related reduction of physical activity on skeletal muscle, possibly naturally aged female mice could be a more suitable model compared to male mice.

Furthermore, we previously found consistently in GEO datasets originating from different studies, that more genes are regulated in old vs. young female muscle tissue compared to old vs. young male muscle tissue [9]. It was striking to find that this sex-difference in number of genes regulated in old vs. young muscle tissue was reversed in mice compared to humans. The higher number of genes regulated during aging in female humans could perhaps be explained by the menopause, which is frequently mentioned as a possible source for sex-differences in aging in humans. Female humans experience a sudden drop in circulating estrogen levels, which has been reported to correlate with several parameters affected by aging, such as energy metabolism [34]. However, it cannot be assumed that this is recapitulated by female mice, since the menopause as known to occur in female humans, does not occur in mice, since ovarian function decreases slowly throughout aging in female mice, as does the secretion of estrogen [35–37]. A sudden menopause as observed in female humans, can be introduced in female mouse models by, for example, ovariectomy [34], but it does not occur naturally. Consequently, this might then explain as well why we did not find a higher number of genes regulated in female mice compared to male mice, as was observed in humans [9]. Besides this, it remains unclear why we found a consistently higher number of regulated genes in male compared to female mice. This knowledge gap cannot be answered yet and remains to be elucidated.

Another interesting sex difference in the muscle-aging trajectory of mice was that in male mice the skeletal muscle transcriptome changed considerably in the oldest group (25 months). The difference between the group prior to this timepoint (the 21 months group) is only 4 months, yet has major significant effects, as an additional 2791 genes were regulated at 25 months compared to the 21 months old group. Noticeably, the top 10 regulated pathways at the age of 25 months were many well-known aging related pathways, such as ‘Sirtuin Signaling Pathway’, ‘Oxidative phosphorylation’ and the ‘Senescence Pathway’ and concomitantly, aging related loss of muscle mass became apparent in the quadriceps muscle at the same timepoint. In addition, the percentage of type I myofibers was significantly higher in the soleus muscle of the 25 months old mice compared to the younger groups as well. This suggests the existence of a tipping point, after which major novel transcriptional changes occur, which did not yet occur at earlier aged groups (17 and 21 months). This finding is in line with another study that has also reported distinctive changes during the late phase of aging in male mice [38]. These results highlight the necessity for careful selection of the exact age being used for a naturally aged mouse model, especially in male mice. Contrastingly, in female mice a more consistent effect of aging on the muscle transcriptome was observed, which could perhaps partially be explained by the fact that in female mice a slow and consistent loss of circulating estrogen occurs [35–37]. In addition, the more prominent role of aging related reduction of physical activity in female mice might also explain this sexual dimorphism. Possibly, a tipping point is apparent in female mice at an earlier or later age or aging is female mice is more gradual without an evident tipping point. The use of earlier timepoints can shed a light on this, but we doubt whether implementing even older mice is desirable, due to a possibly high loss of mice resulting in a lack of statistical power, and a higher likelihood for confounding effects due to aging-related comorbidities.

Noticeably, the quadricep muscle was the only muscle group to display a statistically significant aging related loss of muscle mass. This was in contrast to lean body mass, which, unlike humans, was higher in the aged groups compared to the young group and was maintained at even an old age, as was also found in other studies [39]. Moreover, this does not necessarily mean that aging-related loss of muscle mass does not occur in other muscle groups besides the quadriceps, since it is likely that if more mice, or a slightly older young control group (with perhaps more muscle mass) were included, statistically significant differences would have been observed in the gastrocnemius muscle as well. However, the current results still suggest that the quadriceps muscle group is most susceptible for aging-related loss of muscle mass. This observation is in line with a recent finding in male humans, in which the quadriceps femoris showed the largest aging related loss of muscle volume compared to other muscles [40]. In the latter study, a smaller aging related loss of muscle volume was observed in the lower leg muscles. Although we measured muscle mass and not volume, this notion is in line with what we observed in the tibialis anterior muscles of aged mice. Moreover, another study implementing near-infrared spectrometry to measure mitochondrial function found an aging-related loss of mitochondrial capacity in quadriceps and gastrocnemius muscle, but not in tibialis anterior muscles [41]. Together these studies indicate muscle-specific effects of aging, and support our finding that upper leg muscles are the most susceptible for the effects of aging.

An often overlooked issue with using the naturally aged mouse model are aging related comorbidities. In the current study, the 25 months old groups were inspected, after the isolation of muscle tissue, upon pathology on a macroscopic level. If deviations from normal morphology were detected in other tissue besides muscle, then these tissues were isolated and histopathology was assessed by a board certified pathologist. Such tissues were collected from 87% of the 25 months old mice. Among others, enlarged lymph nodes, livers, spleens, kidneys and pancreata were found, and lymphomas or hydronephrosis (in case of kidneys), were frequently identified to be the underlying cause of these swellings (Suppl. Figure 4). Such comorbidities are not a surprise and are common in aged mice [12], however, these are frequently not investigated or mentioned in publications related to muscle-aging. All in all, these are factors that should be taken into account whilst choosing to use the naturally aged mouse as a model. Alternatively, other ways for the induction of muscle-atrophy can be considered, such as immobilization or restriction of the diet, which have been shown to induce pathways that are also regulated during muscle-aging in humans [42].

Limitations of the current study were that we used one young control group. Slightly older young control groups (e.g. 8 months) might have revealed additional significant effects in, for example, loss of muscle mass. Another limitation of this study is that most molecular analyses (e.g. RNA-seq) were performed in quadriceps muscle, and the observed effects of aging in this muscle might not be representative for the effects of aging in other muscles. This is also reflected by our finding that the quadriceps muscle displayed aging-related loss of muscle mass, but such atrophic effects of aging were not found in e.g. the tibialis anterior muscle. In addition, aging-trajectories could differ between muscles as well due to, e.g., different myofiber type compositions (e.g. soleus muscle), which could be an important factor to take into account when comparisons are made between mice and humans [43]. Lastly, in future research muscle function could be evaluated by performing muscle-specific measurements of force (e.g. maximal isometric torque) as well.

In conclusion, we have identified several sex-specific effects of aging on mice and their skeletal muscles. Aging-related reduction of movement speed is more pronounced in female mice, and is also associated to a larger degree to the aging related changes observed in the quadriceps muscle transcriptome. In male, but not in female mice, aging affects the muscle transcriptome distinctively at a late age (25 months). Furthermore, contrasting to humans, in the skeletal muscles of male mice more genes are regulated compared to female mice. Our results show that the translational value of mice for human muscle-aging, especially in regards to sex-differences, should not be assumed, and that careful selection of the sex and age of mice is required prior to performing such a study. The results of the current study provide novel insights into sex differences in muscle-aging in mice, and their translatability for human muscle-aging, which may aid in the effective implementation of mouse-models in the field of muscle-aging.

### Supplementary Information

Below is the link to the electronic supplementary material.

Supplementary file1 (JPG 2621 KB)

Supplementary file2 (JPG 2706 KB)

Supplementary file3 (JPG 1940 KB)

Supplementary file4 (JPG 6497 KB)

Supplementary file5 (DOCX 15 KB)

## Data Availability

Data are available from the corresponding author upon reasonable request.
