# Supplementary material for: Translatability of mouse muscle-aging for humans: the role of sex
Source: GeroScience. 2024 Jan 24;46(3):3341–60. doi: 10.1007/s11357-024-01082-7 (PMC11009184; doi:10.1007/s11357-024-01082-7)
Supplement: Supplementary file 5 — Supplementary file5 (DOCX 15 KB) [file 11357_2024_1082_MOESM5_ESM.docx]

| **Data other than RNA-seq data** | **Males** | **Females** |
| --- | --- | --- |
| Figure 1A | Kruskal-Wallis + Mann-Whitney U test | Tukey’s HSD test |
| Figure 1B | Kruskal-Wallis + Mann-Whitney U test | Tukey’s HSD test |
| Figure 1C | Tukey’s HSD test | Kruskal-Wallis + Mann-Whitney U test |
| Figure 1D | Kruskal-Wallis + Mann-Whitney U test | Tukey’s HSD test |
| Figure 1E | Kruskal-Wallis + Mann-Whitney U test | Kruskal-Wallis + Mann-Whitney U test |
| Figure 1F | Tukey’s HSD test | Kruskal-Wallis + Mann-Whitney U test |
| Figure 2A | Tukey’s HSD test | Kruskal-Wallis + Mann-Whitney U test |
| Figure 2B | Tukey’s HSD test | Kruskal-Wallis + Mann-Whitney U test |
| Figure 2C | Tukey’s HSD test | Kruskal-Wallis + Mann-Whitney U test |
| Figure 2D | Tukey’s HSD test | Kruskal-Wallis + Mann-Whitney U test |
| Figure 3C | Kruskal-Wallis + Mann-Whitney U test | Tukey’s HSD test |
| Figure 3D | Tukey’s HSD test | Kruskal-Wallis + Mann-Whitney U test |
| Figure 3E | Tukey’s HSD test | Kruskal-Wallis + Mann-Whitney U test |
| Figure 3F | Kruskal-Wallis + Mann-Whitney U test | Kruskal-Wallis + Mann-Whitney U test |
| Figure 3G | Tukey’s HSD test | Tukey’s HSD test |
| Figure 3H | Tukey’s HSD test | Tukey’s HSD test |
| Figure 3J | Tukey’s HSD test | Kruskal-Wallis + Mann-Whitney U test |
| Suppl. Fig. 1A | Tukey’s HSD test | Kruskal-Wallis + Mann-Whitney U test |
| Suppl. Fig. 1B | Kruskal-Wallis + Mann-Whitney U test | Tukey’s HSD test |
| Suppl. Fig. 1C | Tukey’s HSD test | Kruskal-Wallis + Mann-Whitney U test |
| Suppl. Fig. 1D | Kruskal-Wallis + Mann-Whitney U test | Kruskal-Wallis + Mann-Whitney U test |
| Suppl. Fig. 1E | Kruskal-Wallis + Mann-Whitney U test | Kruskal-Wallis + Mann-Whitney U test |
